# Successful separation of male pygopagus with anal canal and urethral reconstruction: a case report

**DOI:** 10.1186/s40792-022-01398-6

**Published:** 2022-03-16

**Authors:** Chiyoshi Toyama, Motonari Nomura, Yuko Tazuke, Chisato Yokota, Naoki Kagawa, Haruhiko Kishima, Akihiro Yoshimura, Takeshi Ujike, Akira Nagahara, Norio Nonomura, Tateki Kubo, Futoshi Matsui, Fumi Matsumoto, Hiroomi Okuyama

**Affiliations:** 1 Department of Pediatric Surgery, Osaka University Graduate School of Medicine, Yamadaoka 2-2, Suita, Osaka 565-0871 Japan; 2 Department of Neurosurgery, Osaka University Graduate School of Medicine, Yamadaoka 2-2, Suita, Osaka 565-0871 Japan; 3 Department of Urology, Osaka University Graduate School of Medicine, Yamadaoka 2-2, Suita, Osaka 565-0871 Japan; 4 Department of Plastic Surgery, Osaka University Graduate School of Medicine, Yamadaoka 2-2, Suita, Osaka 565-0871 Japan; 5 Department of Pediatric Urology, Osaka Women’s and Children’s Hospital, Murodo-Cho 840, Izumi, Osaka 594-1101 Japan

**Keywords:** Conjoined twin, Pygopagus, Anal canal

## Abstract

**Background:**

Pygopagus is a type of conjoined twin binding at the buttocks. Some cases of pygopagus involve the fusion of the gastrointestinal tract, urinary tract, and spinal cord. Few cases of male pygopagus have been reported; however, the prognosis after separation is unclear. Herein, we report a case of male pygopagus in which successful separation was performed with the reconstruction of the anal canal.

**Case presentation:**

Twins with male pygopagus were born at 35 weeks by cesarean section. They shared a common anus, penis, and scrotum with four testes. The infants had normal defecation and urination after birth. The separation surgery was scheduled when they were 5 months. Two distinct anesthesia teams and four surgical teams (neurosurgery, pediatric urology, plastic surgery, and pediatric surgery) were involved in the multidisciplinary approach. After separating the spinal cord, we found that the anal canal and sphincter muscle complex were fused near the anal aperture, and we separated them. The fused penis and testis were separated and reconstructed using the same incisional line as the other separation, and the reconstructions of the anal canals with the sphincter muscle complex were completed. Both patients had an uneventful postoperative course. At 2 years of age, they could walk and defecate independently. In addition, they voided spontaneously without urinary incontinence at the time of 3 years and 11 months.

**Conclusions:**

Separation of the spinal cord with anal canal and urethral reconstruction is important for male pygopagus patients as it allows them to preserve their independent function.

## Background

Conjoined twins occur at a rate of approximately 1 in 50,000–200,000 deliveries, with a female-to-male ratio of approximately 1:3. Pygopagus is a twin type of binding at the buttocks and accounts for approximately 20% of all conjoined twins [1–3]. In pygopagus patients, 6–19% of all cases have connected gastrointestinal tract, pelvis, urinary tract, and nerves [3–5]. Patients with male pygopagus have rarely been reported [1]. The survival rate of all pygopagus is good; however, poor prognosis in terms of motor functions of the lower limbs, defecation, and urination disorders has been reported [3, 6]. The functional prognosis for male pygopagus patients is unknown.

There are various types of bowel fusion in pygopagus, including fusion from the small intestine to the anus or only anal fusion [7]. Almost all of these cases have a fused anal canal; however, there are few detailed reports on the sphincter muscle complex and puborectalis. Separation and reconstruction of the fused organs, including the anal canal, spinal cord, and urogenital organs, must be performed while preserving their postoperative functions [8]. Therefore, surgery should be precise and be conducted with a multidisciplinary approach by surgeons and anesthesiologists with abundant experience in their fields.

Herein, we report a case of successful separation surgery in male pygopagus with multimodality surgical teams.

## Case presentation

A 34-year-old woman, who conceived via in vitro fertilization, received the diagnosis following fetal ultrasound screening at 11 weeks and confirmed by magnetic resornance image (MRI) at 16 weeks of pregnancy. The images revealed the connection between the sacrum and a continuous spinal canal; however, they had separate intra-abdominal organs.

**Fig. 1:**
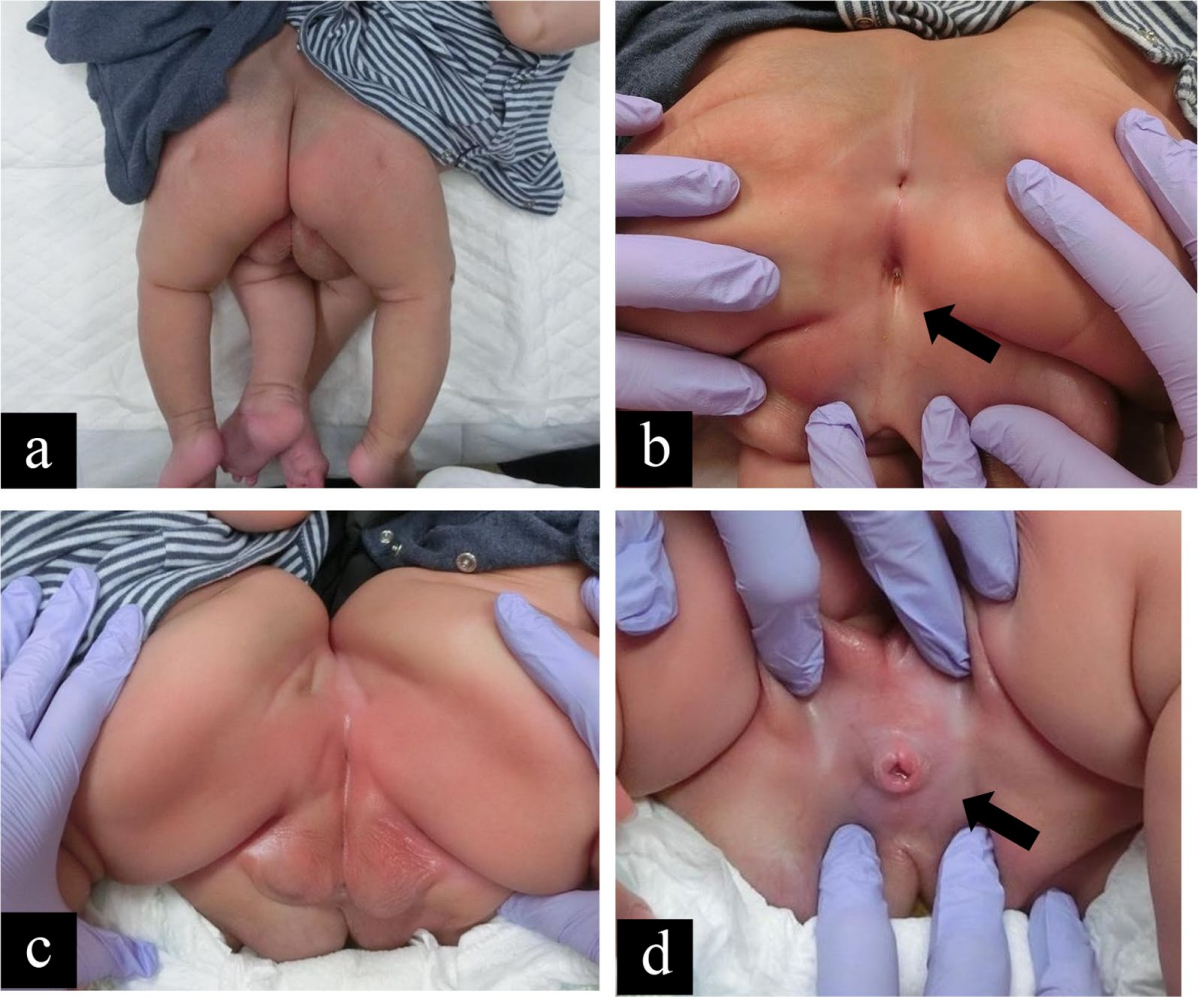
Physical exams before the twins underwent surgical separation. **a** General appearance, **b** fused anus with one anal aperture, **c** fused scrotum with four testes, **d** fused penis with one urethra

**Fig. 2:**
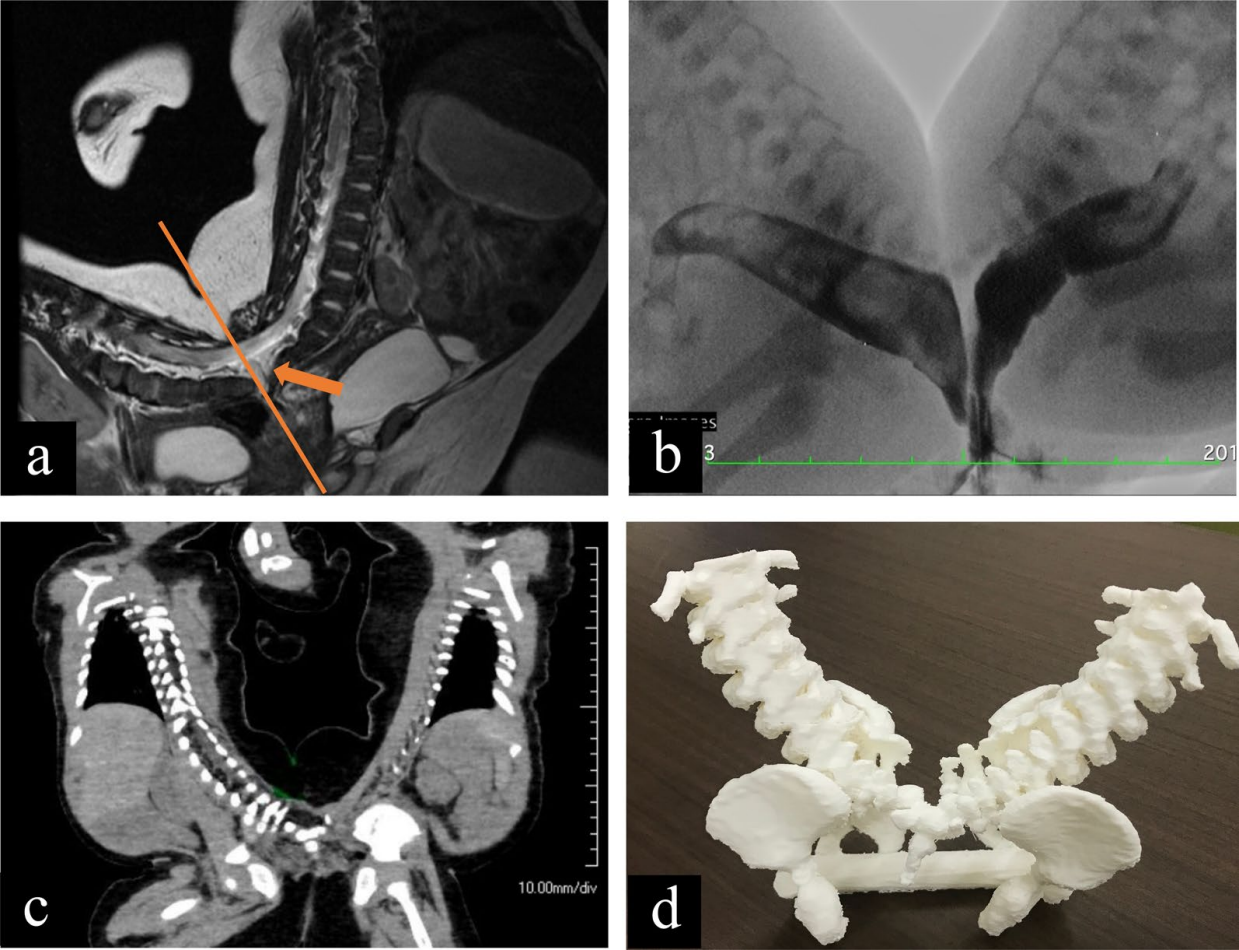
Preoperative image findings and the 3D model. Preoperative image of **a** a fused spinal cord detected by MRI (arrow), **b** separated rectums detected by a gastro-graphin enema. **c** Other intra-abdominal organs have no connection, **d** the 3D model was created for multidisciplinary discussion

Twins with male pygopagus were born at 35 weeks by cesarean section, with a combined birth weight of 4690 g. At birth, the infants had no signs of distress. They shared one anus, one penis, and one scrotum with four testes (Fig. 1a–d, respectively). Defecation and urination after birth were normal. MRI showed that the twins had a fused sacrum, and their spinal cords shared one continuous tube (Fig. 2a). They had a short common anus and rectum (Fig. 2b). Computed tomography (CT) revealed two bladders, rectums, livers, and spleens (Fig. 2c). Additional evaluation of urinary tract showed that twin A had horseshoe kidney and twin B had unilateral vesicoureteral reflux (grade 3). Separation was scheduled when the infants were 5 months. The surgery aimed to separate the sacrum, spinal canal, fused rectum, fused penis, and scrotum while preserving their functions.

Two different anesthesia teams and four surgical teams (neurosurgery, pediatric urology, plastic surgery, and pediatric surgery) were involved. Detailed surgical procedures were discussed twice using a preoperative 3D model (Fig. 2d).

In the operating room, the twins were first intubated, and central venous catheters were inserted by the respective anesthesiologists. The placement of the anesthetic instruments was designed to enable preparation for intraoperative repositioning and post-separation position, as reported in detail by Sato et al. [9]. Urinary catheters were placed into each twin’s bladder using cystourethrography under general anesthesia. Each bladder was not trabeculated, and bladder neck was closed. Common urethra was not wide and extended to the external urethral meatus. We planned the separation beginning from the superior organ toward the inferior organ to avoid contamination due to rectal and penis separation.

**Fig. 3:**
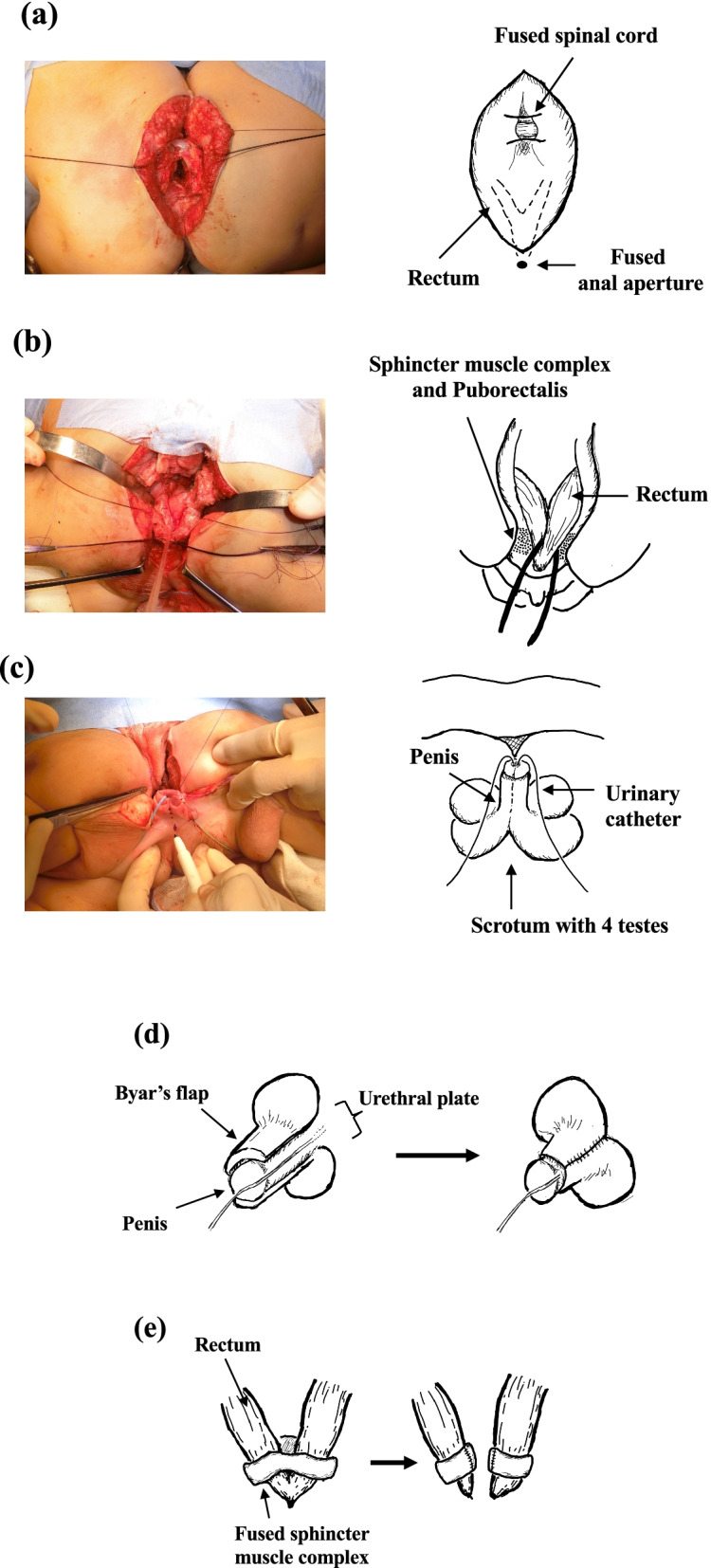
Separation with anal canal and urethral reconstruction. Operative findings: **a** fused spinal cord, **b** fused anus, **c** fused penis. **d** The fused penis and urethra were divided into two pairs of bodies, and the urethral plate and penile skin were sutured with Byar’s flap to wrap the penises. **e** The sphincter muscle complex and puborectalis were fused partially and reconstructed, giving each patient one-half of the sphincter muscles

The patients were placed in the prone position. After the sacrum was separated, a fused nerve root was confirmed within the spinal canal (Fig. 3a). Neurosurgeons separated the fused spinal cord and nerve roots using microscopy and neuro-physical intraoperative monitoring and repaired the dura mater. Details of the surgical separation of conjoined spinal cords of this case have been reported by Yokota et al. [10]. After spinal cord separation, we detected the fusion of the two rectums near the anal aperture (Fig. 3b). Their sphincters and puborectalis were partially shared; therefore, they were separated using an anal stimulator with keen attention to their morphology. Similarly, the fused penises and urethra were separated (Fig. 3c). Four cavernous bodies were detected in the fused penis, which were divided into two pairs of bodies. The pendulous urethrae were separated equally between both infants and peeled off to the bifurcation of the bulbar urethrae. After confirming that the spermatic cords of both infants were not crossed, the scrotums were separated at the same incision line as the skin. Each perineal urethrostomy was created, because each separated urethra was not wide enough to perform single stage urethroplasty. The urethral plate and penile skin were sutured with Byar’s flap to wrap the penises. The scrotums were reconstructed with the Dartos fascia, and the skin was closed (Fig. 3d). The dissected rectum and anal canal were reconstructed to pass through the sphincter and puborectalis (Fig. 3e). The position of the neo-anus was detected and placed on the separation line using a muscle stimulator. Finally, a transverse colostomy was performed to cover the anus.

**Fig. 4:**
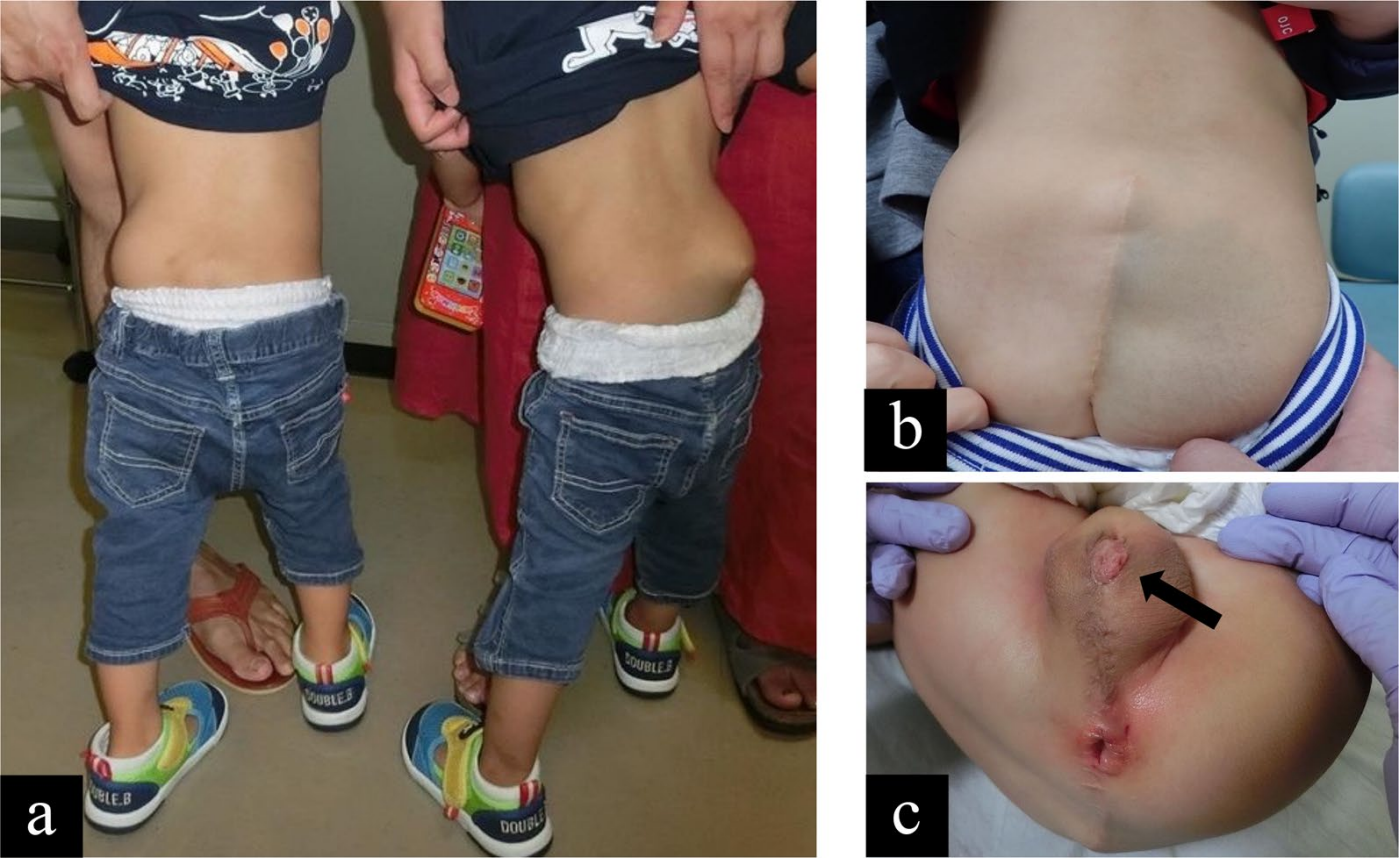
General appearance after separation of the pygopagus patient. Follow-up physical examination: **a** the patients stand by themselves. **b** Their scars are clear, and **c** external urethral orifice (arrow) and anal aperture are shown

Both patients had an uneventful postoperative course. They had no motor dysfunction of the lower limb and no obvious urinary incontinence, and they underwent a stoma closure surgery 4 months later. Nine months later, they underwent tubularized incised plate urethroplasty for hypospadias. Eighteen months after separation, both children could walk alone (Fig. 4a). The scars were clear, without complications (Fig. 4b), and external urethral orifices and anal apertures were confirmed (Fig. 4c). They had bowel movements once daily by enema without soiling. They voided spontaneously without urinary incontinence at the time of 3 years and 11 months.

## Discussion

We reported a rare case of male pygopagus who underwent successful separation surgery with anal canal and urethral reconstruction following a multidisciplinary approach. The infants had preserved defecation, urination, and lower limb function.

Male pygopagus is very rare, and well-documented cases are shown in Table 1 [1, 7, 11–13]. In almost all cases, the intestines and urinary tracts were connected, and two of five cases had neural connections. Although most cases did not describe the postoperative function in detail, two cases, including our case, had no functional impairment or lower limb paralysis.

**Table 1:** Male pygopagus conjoined twins

	Author (year)	Operative timing	Age at operation	Shared organ			Outcome	Urinaryfunction	Defecation
				Spinal cord	Intestine	Urinary tract			
1	Luce (1956)	Elective	17 days	Dural sac	NA	NA	Both survived	NA	NA
2	Ventura (1998)	Elective	2 days	No	No	No	Both survived	NA	NA
3	Leelanukrom (2004)	Elective	6 months	NA	Rectum Anus	External Genitalia	Both survived	NA	NA
4	Gohary (2009)	Elective	19 months	Dural sac	Rectum Anus	External Genitalia	Both survived	A: Good B: Neuropathic bladder	A: GoodB: Soiling at night
5	Saguil (2009)	Emergent	2 days	NA	Rectum Anus	External Genitalia	Both dead	–	–
6	Our cases	Elective	6 months	Dural sac	Anus	External Genitalia	Both survived	A: GoodB: Good	A: GoodB: Good

*NA* not available

Reconstruction of the anal canal and urethra in a pygopagus patient was only reported by Liu et al. [9] in a case, wherein the sphincter muscle in a female pygopagus patient could contract normally and could be considered the normal sphincter muscle. The surgeon incised the common anal canal longitudinally through the middle to give each baby half of the sphincter muscles. Both patients were continent and had no constipation or soiling problems. In our cases, a similar reconstruction of the sphincter muscle was performed with a male pygopagus condition, and good prognoses were achieved. Although there are few previous reports on urinary tract reconstruction, we performed a reconstruction using Byar’s flap method, and our patients had no obvious dysuria. We conclude that long-term follow-up of our patients is necessary.

Can et al. reported the usefulness of a multidisciplinary approach for treating female pygopagus in Vietnam [14^1^]. Our treatment approach was also similar as several meetings were conducted preoperatively regarding the operation with experienced surgeons and anesthesiologists. Detailed surgical procedures were simulated by multidisciplinary teams using a 3D model. Therefore, we successfully separated the conjoined twins without any serious complications. Postoperative functions of the lower extremities, including urination and defecation, were preserved.

Precise evaluation and surgical technique for fused organs in male pygopagus patients are important for successful morphological and functional separation.

## Conclusions

Surgery involving anal canal and urethral reconstruction using a multidisciplinary approach is important for male pygopagus patients as it enables preservation of postoperative functions.

## Data Availability

Not applicable.

## Abbreviations

MRI: Magnetic resonance imaging
CT: Computed tomography
NA: Not available
